# Role of EphA2 in host defense against oro-pharyngeal candidiasis

**DOI:** 10.1080/20002297.2020.1711619

**Published:** 2020-01-09

**Authors:** Ingar Olsen

**Affiliations:** Department of Oral Biology, Faculty of Dentistry, University of Oslo, Oslo, Norway

**Keywords:** Oral epithelial cells, neutrophils, oral candidiasis, commensals, pathogens

## Abstract

Oral defense should be able to sense the burden of, and distinguish between fungal commensals and pathogens, so that an adequate inflammatory response can be set up. Recently, Ephrin type-A receptor 2 (EphA2) was identified on oral epithelial cells and neutrophils that recognizes *Candida albicans* and induces adaptive protective host responses against this organism. The studies have increased our knowledge of how epithelial cells and neutrophils contribute to host defense against oral yeast infection.

The oral cavity is continuously exposed to a heterogeneous microbiota consisting of bacteria, virus and yeasts interacting with epithelial cells. A vast number of fungal species (up to 100) have been detected in the oral cavity by molecular methods over the last years [1–3]. It is clear that *Candida*, and in particular *Candida albicans*, is a true inhabitant of the oral cavity in many of us [4], but *C. albicans* can become pathogenic in vulnerable patients. Patients with neutropenia or diabetes mellitus frequently suffer from candidiasis [5]. It is also a common infection of the oral mucosa in babies, denture wearers and patients with dry mouth [6–8]. When becoming invasive, candidiasis has a mortality rate of 40–50% [5]. One common example of *Candida* infection in the dental area is oro-pharyngeal candidiasis that can be the cause of significant morbidity, particularly in immunocompromised patients or in individuals with defects in T-cell immunity such as HIV/AIDS.

*C. albicans* appears morphologically as ovoid yeast cells or pseudohyphae. The yeast form has a low capacity to invade tissue and initiate the release of proinflammatory cytokines and defense peptides, whereas hyphae are more invasive and strongly stimulate the production of mucous, cytokines, chemokines and anti-microbial peptides [4,9–11]. Unfortunately, our knowledge about the interaction between *C. albicans* and mucosal and other host cells is limited and needs to be extended. The purpose of this editorial is to draw attention to a recently recognized pattern recognition receptor (PRR) on the oral mucosa and neutrophils and to discuss its significance in distinguishing between commensalism and pathogenicity of oral yeasts.

*Candida* cells on the mucosa can be rrecognized by PRRs which identify a number of pathogen-associated patterns (PAMPs) expressed on invading organisms [12–14]. PAMP-PRR interactions on myeloid cells elicit mechanisms intended to neutralize the pathogen such as phagocytosis, respiratory burst and immune mediator release [15,16]. Other major classical fungal PRRs on myeloid cells include C-type lectin-receptors (e.g. dectin-1) and Toll-like-, NOD-like- and RIG-I-like receptors (for a review see [16]). During the invasion of the epithelial cell lining in the oro-pharynx, *C. albicans* produces invasins that activate E-cadherin and the epidermal growth factors EGFR and HER2 on epithelial cells [17]. These non-classical receptors can bind *Candida* hyphae [5,18,19]. Invasion of oral epithelial cells is promoted by candidalysin which is a small toxin secreted by hyphae [20]. In the host response to *Candia* infection, the role of IL-17 should also be mentioned as its significance has been demonstrated in a number of studies from both humans and mice [21]. IL-17, which is generated under *Candida* infection, modulates pro-inflammatory cytokines, chemokines and antimicrobial proteins to protect the host via the influx of neutrophils and candicidal activities [22].

Oral epithelial cells should be able to distinguish between fungal commensals and pathogens so that an adequate inflammatory response can be elicited. Recently, Swidergall et al. [23,24] identified Ephrin type-A receptor 2 (EphA2) on oral epithelial cells and neutrophils that detect *C. albicans* and induces adaptive protective host responses against this organism.

Eph receptors constitute the largest family of Receptor Tyrosine Kinases that contain a single membrane-spanning segment [25]. The Eph receptor tyrosine kinases and their ephrin ligands serve as critical regulators of cell contact-dependent signaling and patterning [26] and participate in several types of cell-cell communications [25]. They can affect diverse biological reactions varying from adhesion to repulsion, and increased versus decreased motility.

Swidergall et al. [23] found that the EphA2 receptor on oral epithelial cells acts as a PRR that binds to exposed β-glucans on the surface of *C. albicans*. Activation of the receptor was indicated by phosphorylation of the EphA2 and accumulation of the receptor around yeast cells. Activation was required for uptake and damage to yeasts by oral epithelial cells. EphA2 has previously been shown to interact with EFGR needed for endocytosis of *C. albicans* [27]. Swidergall et al. [23] also observed that *C. albicans* caused interdependent and reciprocal co-activation of EphA2 and EGFR. Activation of both receptors was needed for efficient fungal-induced endocytosis.

The binding to EphA2 activated signal transducer activator of transcription 3 (STAT3), mitogen-activated kinase regulated kinase 1/2 and p38 signaling in an inoculum-dependent manner [23]. These actions secured the initiation of a pro-inflammatory and antifungal response. Sustained intracellular signaling by EphA2 required interaction with live yeast cells. In epithelial cells exposed to *C. albicans* also nuclear factor-kappa B (NF-κB) was activated. However, this was also achieved by dectin-1 in response to proliferating *Candida*. Dectin-1 was originally thought to function primarily on myeloid cells [28] and is a major receptor in antifungal immunity. The outgrowth of yeasts in the oral cavity caused strong receptor activation and downstream signaling through signal inducer and STAT3, as well as mitogen-activated protein kinases (MAPKs) that activate a distinct profile of chemokines, cytokines and host defense peptides [16]. Thus, activation of STAT3 stimulated expression of inflammatory molecules such as CCL20, CCL13 and S100A8 and secretion of human β-defensin 2. Also, IL-α, IL-1β and IL-8 were expressed, but independent of STAT3. This was supported by findings from EphA2 knockout mice with acute oro-pharyngeal candidiasis, which had a reduced antifungal cytokine and chemokine response in the tongue leading to a higher fungal burden and more severe disease [23].

Accordingly, the study clearly showed that Eph2A served as a PRR for β-glucans able to sense the epithelial cell burden of harboring yeasts that might cause disseminated infection. The Eph2A receptor was also needed for a maximal host inflammatory response to *C. albicans*. The yeast and mycelial form induced similar activation of EphA2. Notably, EphA2 could also be stimulated by other fungal species such as *Candida glabrata, Aspergillus fumigatus* and *Rhizopus delemar* but not by bacteria like *Escherichia coli* and *Staphylococcus aureus*. EphA2 has also been found to recognize *Plasmodium, Chlamydia trachomatis*, and several viruses (for a review see [24]). β-glucan but not mannan was the only yeast ligand recognized by EphA2. It was also noteworthy that activation of EphA2 and the induced inflammatory response correlated directly with the number of yeast cells. This indicated that oral epithelial cells could estimate the quantity of colonizing yeast cells in a receptor-dependent way. Epithelial β-glucan sensing distinguished between fungal colonization and overgrowth, thereby providing protective immunity in the oral cavity. Following fungal overgrowth, epithelial cells secreted cytokines and chemokines and attracted phagocytes to the infection site.

In another recent study, Swidergall et al. [24] examined whether EphA2 located on neutrophils were able to stimulate antifungal activity against oro-pharyngeal yeast infection. While oral epithelial cells sensed β-glucan through EphA2 and dectin-1, neutrophils recognized β-glucan via dectin-1 and complement receptor 3 (CR3; CD11b/CD18) [16,24,29,30]. In addition, neutrophils, recruited to the site of infection, secreted cytokines and chemokines, as also shown previously [14,31]. Swidergall et al. [24] found that *EphA2^−/-^* mice had delayed infiltration of neutrophils and inflammatory monocytes in the yeast-infected oral cavity. This led to a more severe disease than observed in wild-type mice. The authors found that EphA2 together with Fcɣ receptors (FcɣRs) were required for neutrophils to restrict the proliferation of *C. albicans* in oro-pharyngeal candidiasis. Actually, Fcɣ was needed for neutrophils to kill *C. albicans in vitro*, and mice that lacked the FcɣR1 were highly susceptible to oro-pharyngeal candidiasis. It has previously been shown that blocking of FcɣR prevents the ability of neutrophils to inhibit *C. albicans in vitro* [32].

Accordingly, EphA2 on neutrophils acted as a PRR for β-glucans that augmented Fcɣ-mediated antifungal activity important for controlling early fungal proliferation in oro-pharyngeal candidiasis. It co-operated with Fcɣ to augment ERK1/2 phosphorylation that increased priming of p47^phox^ (the phagocyte NADPH oxidase/NOX_2_ organizer). This again led to increased cellular production of reactive oxygen species (ROS) and neutrophil killing of opsonized *C. albicans*. EphA2 recognition of fungal β-glucans was required for p47^phox^ priming and generation of fungicidal levels of ROS in response to ligation to Fcɣ [24].

The fact that EphA2 increased FcɣR- mediated activity during infection with *C. albicans* suggested that this protein could extend the response of myeloid cells to involve also other microbial pathogens. An unexpected effect was reported with the opportunistic pathogen *Cryptococcus neoformans*, which promoted EphA2 activity via CD44, and this, in turn, created a permeable barrier that facilitated the migration of *C. neoformans* across the human blood-brain barrier [33].

The studies by Swidergall et al. [23,24] have increased our knowledge on how epithelial cells and neutrophils act to distinguish between commensal and pathogenic fungi in the oral cavity, and how they use a specific receptor to achieve this. They have demonstrated that EphA2 functions as a PRR for β-glucans that senses epithelial cell fungal burden and is needed for the maximal mucosal inflammatory response to *C. albicans*. In neutrophils, this receptor augments the Fcγ receptor-mediated antifungal activity and controls early fungal proliferation during oro-pharyngeal candidiasis. The studies have also confirmed the importance of epithelial cells and neutrophils in the local and systemic defense against oral fungal infection.

As often in research, new findings raise new questions, some of which have been expressed by Dambuza and Brown [8]: Are other fungal factors needed to sustain EphA2 signaling and to induce inflammatory responses? What effect could the interaction with EphA2 have on other receptors, e.g. EGFR-involved immune responses? Can candidalysin circumvent this receptor? Is there a role for EphA2 in other mucosal sites, and how does EphA2 influence T-helper-17 adaptive immunity, important for controlling fungal infection? It could be added: Does EphA2 activity prompted via CD44 create a permeable blood-brain barrier for other microorganisms than *C. neoformans*? One may also wish that other oral organisms among fungi and virus could be tested for recognition by phagocytic cells via EphA2.

Having said this, we look with great interest to further achievements in the knowledge on mechanisms of host interaction with candida, both as commensals and pathogens.
